# Supplementary material for: Maternal and paternal employment in agriculture and early childhood development: A cross-sectional analysis of Demographic and Health Survey data
Source: PLOS Glob Public Health. 2023 Jan 6;3(1):e0001116. doi: 10.1371/journal.pgph.0001116 (PMC10021554; doi:10.1371/journal.pgph.0001116)
Supplement: S1 Table — (DOCX) [file pgph.0001116.s001.docx]

**S1 Table** Demographic and Health Surveys (DHS) included in the sample

| **Country** | **Phase** | **Year** | **N** |
| --- | --- | --- | --- |
| Benin | Phase VII | 2017-2018 | 1,387 |
| Burundi | Phase VII | 2016-2017 | 1,547 |
| Cambodia | Phase VII | 2014 | 675 |
| Congo | Phase VI | 2011-2012 | 828 |
| Haiti | Phase VII | 2016-2017 | 824 |
| Rwanda | Phase VII | 2019-2020 | 1,046 |
| Senegal | Phase VIII | 2019 | 450 |
| Togo | Phase VI | 2013-2014 | 799 |
| Uganda | Phase VII | 2016 | 900 |
