# Supplementary material for: Maternal and paternal employment in agriculture and early childhood development: A cross-sectional analysis of Demographic and Health Survey data
Source: PLOS Glob Public Health. 2023 Jan 6;3(1):e0001116. doi: 10.1371/journal.pgph.0001116 (PMC10021554; doi:10.1371/journal.pgph.0001116)
Supplement: S2 Table — (DOCX) [file pgph.0001116.s002.docx]

**S2 Table** Differences between households included in the analytic sample and those excluded by exclusion reason

|  | **Paternal employment status** | | |  | **Missing parental occupation data** | | |
| --- | --- | --- | --- | --- | --- | --- | --- |
|  | Included | Excluded | p-value for difference* |  | Included | Excluded | p-value for difference* |
|  | Employed fathers | Unemployed fathers |  |  | No missing parental occupation data | Missing parental occupation data |  |
| *Household characteristics* |  |  |  |  |  |  |  |
| Size | 6.83±3.73 | 7.47±4.85 | 0.51 |  | 6.81±3.71 | 8.24±4.63 | 0.72 |
| Number of children <5 y | 203.39 | 222.02 | 0.47 |  | 2.03±1.10 | 2.21±1.05 | <0.01 |
| Lives in rural area | 75.53 | 69.1 | 0.37 |  | 75.73 | 63.39 | 0.09 |
| Wealth quintile |  |  |  |  |  |  |  |
| Poorest | 24.21 | 20.7 | 0.45 |  | 24.24 | 22.22 | <0.01 |
| Poorer | 21.6 | 20.43 |  |  | 21.71 | 14.72 |  |
| Middle | 19.96 | 20.19 |  |  | 19.95 | 20.66 |  |
| Richer | 17.42 | 28.11 |  |  | 17.44 | 16.09 |  |
| Richest | 16.82 | 10.57 |  |  | 16.66 | 26.3 |  |
| *Maternal characteristics* |  |  |  |  |  |  |  |
| Age, years | 31.49±6.36 | 32.55±7.16 | 0.31 |  | 31.49±6.37 | 31.42±5.80 | 0.90 |
| Highest level of education |  |  |  |  |  |  |  |
| None | 38.21 | 41.51 | 0.37 |  | 37.87 | 58.69 | <0.01 |
| Primary | 40.37 | 41.42 |  |  | 40.81 | 13.97 |  |
| Secondary or higher | 21.42 | 17.07 |  |  | 21.32 | 27.33 |  |
| *Paternal characteristics* |  |  |  |  |  |  |  |
| Age, years | 37.20±7.84 | 38.56±9.57 | 0.32 |  | 37.16±7.84 | 39.40±8.05 | <0.01 |
| Highest level of education |  |  |  |  |  |  |  |
| None | 28.28 | 29.29 | 0.91 |  | 0.28±0.45 | 0.45±0.50 | <0.01 |
| Primary | 43.08 | 43.67 |  |  | 43.54 | 15.86 |  |
| Secondary or higher | 28.64 | 27.04 |  |  | 28.46 | 39.28 |  |
| *Child characteristics* |  |  |  |  |  |  |  |
| Male | 50.74 | 54.29 | 0.57 |  | 50.83 | 45.59 | 0.26 |
| Age, months | 46.61±7.11 | 48.19±6.87 | 0.08 |  | 46.61±7.11 | 46.63±7.11 | 0.98 |
| Overall development on-track | 60.12 | 52.91 | 0.25 |  | 59.98 | 68.57 | 0.07 |
| Early Childhood Development Index Score (range 0-10) | 5.30±1.76 | 5.14±1.69 | 0.43 |  | 5.30±1.76 | 5.48±1.71 | 0.30 |
| *Childcare practices* |  |  |  |  |  |  |  |
| Number of stimulation activities provided by |  |  |  |  |  |  |  |
| Mother | 1.77±1.77 | 1.58±1.63 | 0.39 |  | 1.77±1.77 | 1.49±1.77 | 0.14 |
| Father | 0.83±1.43 | 0.48±0.88 | <0.01 |  | 0.83±1.44 | 0.80±1.35 | 0.87 |
| Other household members | 1.69±1.96 | 1.63±1.86 | 0.81 |  | 1.69±1.96 | 1.73±1.82 | 0.84 |
| Child not left alone for >1 hour in the past week | 81.69 | 84.53 | 0.59 |  | 81.64 | 84.58 | 0.39 |
| Child not left with another child for >1 hour in the past week | 71.25 | 71.54 | 0.96 |  | 71.20 | 74.10 | 0.55 |
| Child provided adequate supervision | 64.65 | 68.70 | 0.49 |  | 64.56 | 70.24 | 0.23 |
| Child attended an early childhood education programme | 24.44 | 24.77 | 0.96 |  | 24.44 | 24.85 | 0.93 |
| *Women’s empowerment* |  |  |  |  |  |  |  |
| Access to and control over resources | -0.05±0.92 | -0.14±0.91 | 0.44 |  | -0.04±0.92 | -0.25±1.01 | 0.08 |
| Decision-making | 0.37±0.69 | 0.27±0.73 | 0.36 |  | 0.38±0.69 | -0.15±0.90 | <0.01 |
| Attitudes towards wife-beating | -1.21±1.65 | -1.19±1.96 | 0.93 |  | -1.21±1.64 | -1.31±1.85 | 0.66 |
| Total empowerment | -0.89±2.23 | -1.06±2.83 | 0.69 |  | -0.88±2.21 | -1.70±2.97 | 0.02 |

* Based on a Wald test for differences across groups.
