# Supplementary material for: Maternal and paternal employment in agriculture and early childhood development: A cross-sectional analysis of Demographic and Health Survey data
Source: PLOS Glob Public Health. 2023 Jan 6;3(1):e0001116. doi: 10.1371/journal.pgph.0001116 (PMC10021554; doi:10.1371/journal.pgph.0001116)
Supplement: S3 Table — (DOCX) [file pgph.0001116.s003.docx]

**S3 Table** Child, maternal, paternal, and household characteristics by maternal employment and parental occupation

|  |  | **Maternal employment*** | | | **Occupation** | | | | | | |
| --- | --- | --- | --- | --- | --- | --- | --- | --- | --- | --- | --- |
|  | Sample | Unemployed | Employed | | Mother employed in agriculture; father employed in non-agriculture | Mother employed in non-agriculture; father employed in agriculture | | Both parents employed in agriculture | | Both parents employed in non-agriculture | |
| *Household characteristics* |  |  | |  |  | |  | |  | |  |
| Size | 6.81±3.71 | 7.08±4.33 | | 6.76±3.59 | 6.39±3.3 | | 7.26±4.31 | | 6.96±3.52 | | 6.40±3.34 |
| Number of children <5 y | 2.03±1.10 | 2.20±1.22 | | 2.00±1.07 | 2.00±0.99 | | 2.07±1.23 | | 2.09±1.07 | | 1.85±1.00 |
| Lives in rural area | 75.73 | 63.81 | | 77.88 | 91.68 | | 86.8 | | 95.97 | | 45.96 |
| Wealth quintile | 24.24 | 21.49 | | 24.74 | 21.7 | | 33.8 | | 35.85 | | 7.66 |
| Poorest | 21.71 | 20.42 | | 21.94 | 26.14 | | 27.18 | | 27.33 | | 11.15 |
| Poorer | 19.95 | 17.19 | | 20.44 | 21.29 | | 22.08 | | 20.57 | | 19.18 |
| Middle | 17.44 | 18.49 | | 17.25 | 18.43 | | 12.71 | | 13.44 | | 23.77 |
| Richer | 16.66 | 22.42 | | 15.62 | 12.43 | | 4.23 | | 2.81 | | 38.24 |
| *Maternal characteristics* |  |  |  | |  |  | |  | |  | |
| Age, years | 31.49±6.37 | 29.86±6.12 | 31.79±6.37 | | 31.31±6.18 | 32.48±6.49 | | 31.97±6.64 | | 31.40±5.98 | |
| Highest level of education |  |  |  | |  |  | |  | |  | |
| None | 37.87 | 34.62 | 38.45 | | 32.25 | 43.28 | | 49.55 | | 24.62 | |
| Primary | 40.81 | 35.78 | 41.71 | | 53.86 | 43.85 | | 44.12 | | 33.19 | |
| Secondary or higher | 21.32 | 29.60 | 19.83 | | 13.89 | 12.87 | | 6.32 | | 42.19 | |
| *Paternal characteristics* |  |  |  | |  |  | |  | |  | |
| Age, years | 37.16±7.84 | 36.51±7.78 | 37.28±7.84 | | 36.11±7.42 | 38.5±8.23 | | 37.15±8.11 | | 37.27±7.37 | |
| Highest level of education |  |  |  | |  |  | |  | |  | |
| None | 28.00 | 26.47 | 28.28 | | 22.78 | 36.73 | | 36.53 | | 15.96 | |
| Primary | 43.54 | 38.70 | 44.41 | | 54.78 | 44.29 | | 50.94 | | 32.52 | |
| Secondary or higher | 28.46 | 34.82 | 27.31 | | 22.44 | 18.99 | | 12.53 | | 51.52 | |
| *Child characteristics* |  |  |  | |  |  | |  | |  | |
| Male | 50.83 | 49.23 | 51.12 | | 48.78 | 51.80 | | 51.74 | | 50.88 | |
| Age, months | 46.61±7.11 | 46.25±7.02 | 46.68±7.13 | | 46.38±6.92 | 47.06±7.13 | | 47.02±7.16 | | 46.17±7.12 | |
| Overall development on-track | 59.98 | 61.97 | 59.62 | | 59.09 | 63.68 | | 52.04 | | 67.26 | |
| Early Childhood Development Index Score (range 0-10) | 5.30±1.76 | 5.31±1.82 | 5.30±1.75 | | 5.36±1.73 | 5.27±1.67 | | 4.96±1.66 | | 5.71±1.82 | |
| *Childcare practices* |  |  |  | |  |  | |  | |  | |
| Number of stimulation activities provided by |  |  |  | |  |  | |  | |  | |
| Mother | 1.77±1.77 | 1.81±1.90 | 1.76±1.74 | | 1.90±1.65 | 1.60±1.73 | | 1.55±1.60 | | 2.05±1.90 | |
| Father | 0.83±1.44 | 0.77±1.37 | 0.84±1.45 | | 0.86±1.27 | 0.74±1.23 | | 0.68±1.44 | | 1.08±1.58 | |
| Other household members | 1.69±1.96 | 1.55±1.87 | 1.71±1.97 | | 1.64±1.84 | 1.56±1.85 | | 1.80±2.06 | | 1.69±1.96 | |
| Child not left alone for >1 hour in the past week | 81.64 | 84.04 | 81.21 | | 83.26 | 77.14 | | 82.10 | | 81.33 | |
| Child not left with another child for >1 hour in the past week | 71.20 | 80.36 | 69.55 | | 65.35 | 69.59 | | 61.72 | | 80.82 | |
| Child provided adequate supervision | 64.56 | 74.70 | 62.73 | | 60.10 | 61.21 | | 56.67 | | 71.95 | |
| Child attended an early childhood education programme | 24.39 | 28.75 | 23.61 | | 19.51 | 24.89 | | 11.01 | | 40.15 | |
| *Women’s empowerment* |  |  |  | |  |  | |  | |  | |
| Access to and control over resources | -0.04±0.92 | -1.53±0.35 | 0.23±0.72 | | 0.00±0.58 | 0.43±0.79 | | 0.00±0.61 | | 0.49±0.73 | |
| Decision-making | 0.38±0.69 | 0.10±0.81 | 0.43±0.65 | | 0.47±0.65 | 0.40±0.63 | | 0.41±0.69 | | 0.45±0.61 | |
| Attitudes towards wife-beating | -1.21±1.64 | -1.06±1.67 | -1.24±1.64 | | -1.67±1.57 | -0.93±1.71 | | -1.66±1.58 | | -0.71±1.51 | |
| Total empowerment | -0.88±2.21 | -2.49±2.02 | -0.59±2.12 | | -1.2±1.89 | -0.09±2.25 | | -1.25±1.96 | | 0.22±1.99 | |

* All fathers in the sample were employed
