# Supplementary material for: Maternal and paternal employment in agriculture and early childhood development: A cross-sectional analysis of Demographic and Health Survey data
Source: PLOS Glob Public Health. 2023 Jan 6;3(1):e0001116. doi: 10.1371/journal.pgph.0001116 (PMC10021554; doi:10.1371/journal.pgph.0001116)
Supplement: S4 Table — (DOCX) [file pgph.0001116.s004.docx]

**S4 Table** Maternal and paternal occupation type by parental education, household wealth, and household location

|  | Highest level of education* | | | Household wealth | | | | | Household location | |
| --- | --- | --- | --- | --- | --- | --- | --- | --- | --- | --- |
|  | No education | Primary | Secondary or higher | Poorest | Poorer | Middle | Richer | Richest | Urban | Rural |
| *Maternal occupation* |  |  |  |  |  |  |  |  |  |  |
| Agriculture - self-employed | 57.2% | 46.8% | 22.5% | 62.7% | 54.5% | 43.3% | 35.3% | 14.1% | 14.0% | 54.7% |
| Agriculture - employee | 4.7% | 12.1% | 4.4% | 5.8% | 8.6% | 10.6% | 10.4% | 4.4% | 3.5% | 9.0% |
| Professional, technical, or managerial | 0.2% | 0.7% | 16.6% | 28.0% | 0.8% | 1.6% | 3.0% | 17.7% | 9.1% | 2.1% |
| Clerical | 0.0% | 0.1% | 1.0% | 0.0% | 0.0% | 0.0% | 0.1% | 1.4% | 0.7% | 0.1% |
| Sales | 25.1% | 20.9% | 35.6% | 19.0% | 21.8% | 26.8% | 30.9% | 36.1% | 45.0% | 20.2% |
| Household and domestic | 0.3% | 0.3% | 0.5% | 0.1% | 0.1% | 0.7% | 0.3% | 1.0% | 1.1% | 0.1% |
| Services | 4.4% | 5.3% | 8.2% | 1.5% | 3.8% | 5.1% | 8.2% | 14.0% | 12.2% | 3.7% |
| Skilled manual | 4.8% | 7.8% | 8.7% | 4.9% | 5.3% | 7.5% | 9.5% | 9.3% | 10.8% | 5.7% |
| Unskilled manual | 3.4% | 6.0% | 2.5% | 5.8% | 5.0% | 4.3% | 2.3% | 2.1% | 3.6% | 4.5% |
| *Paternal occupation* |  |  |  |  |  |  |  |  |  |  |
| Agriculture - self-employed | 68.6% | 52.3% | 33.2% | 73.9% | 61.0% | 50.6% | 34.4% | 8.9% | 17.6% | 60.4% |
| Agriculture - employee | 5.3% | 11.5% | 2.4% | 6.0% | 7.8% | 9.5% | 9.6% | 3.0% | 3.6% | 8.2% |
| Professional, technical, or managerial | 2.4% | 2.5% | 20.9% | 1.8% | 3.4% | 5.8% | 10.4% | 26.2% | 17.8% | 5.0% |
| Clerical | 0.1% | 13.0% | 1.8% | 0.1% | 36.0% | 22.0% | 0.5% | 2.7% | 1.7% | 0.3% |
| Sales | 4.4% | 4.0% | 8.1% | 1.9% | 3.6% | 4.5% | 7.4% | 13.7% | 11.0% | 3.8% |
| Household and domestic | 0.3% | 0.6% | 0.2% | 0.3% | 0.4% | 0.7% | 0.3% | 0.4% | 0.8% | 0.3% |
| Services | 4.4% | 6.4% | 9.9% | 2.5% | 3.6% | 5.7% | 11.7% | 17.2% | 14.9% | 4.7% |
| Skilled manual | 7.5% | 13.7% | 20.1% | 6.6% | 11.9% | 14.9% | 19.8% | 23.6% | 25.1% | 10.8% |
| Unskilled manual | 7.0% | 9.0% | 3.5% | 6.9% | 7.9% | 8.1% | 6.1% | 4.4% | 7.6% | 6.7% |

* Maternal occupation type tabulated by maternal educational level, and paternal occupation type tabulated by paternal education level
