# Supplementary material for: Maternal and paternal employment in agriculture and early childhood development: A cross-sectional analysis of Demographic and Health Survey data
Source: PLOS Glob Public Health. 2023 Jan 6;3(1):e0001116. doi: 10.1371/journal.pgph.0001116 (PMC10021554; doi:10.1371/journal.pgph.0001116)
Supplement: S5 Table — (DOCX) [file pgph.0001116.s005.docx]

**S5 Table** Associations between parental occupation and development domains among children aged 36-59 months^1^

|  | Early Childhood Development Index Score (range 0-10) | | Cognitive development on-track | | Socio-emotional development on-track | | Literacy-numeracy development on-track | | Physical development on-track | |
| --- | --- | --- | --- | --- | --- | --- | --- | --- | --- | --- |
|  | Unadjusted MD | Adjusted MD | Unadjusted RR | Adjusted RR | Unadjusted RR | Adjusted RR | Unadjusted RR | Adjusted RR | Unadjusted RR | Adjusted RR |
| Both parents employed in non-agriculture | Ref | Ref | Ref | Ref | Ref | Ref | Ref | Ref | Ref | Ref |
| Mother employed in agriculture; father employed in non-agriculture | -0.35  (-0.52, -0.17) | -0.14  (-0.32, 0.04) | 0.90  (0.86, 0.94) | 0.93  (0.88, 0.97) | 1.02  (0.97, 1.08) | 0.94  (0.88, 1.01) | 0.60  (0.47, 0.76) | 0.94  (0.73, 1.20) | 1.02  (0.99, 1.04) | 1.02  (0.99, 1.04) |
| Mother employed in non-agriculture; father employed in agriculture | -0.43  (-0.59, -0.27) | -0.09  (-0.25, 0.07) | 0.95  (0.92, 0.99) | 1.00  (0.96, 1.04) | 1.02  (0.97, 1.08) | 0.99  (0.94, 1.05) | 0.45  (0.36, 0.57) | 0.86  (0.68, 1.10) | 0.98  (0.96, 1.01) | 1.00  (0.97, 1.03) |
| Both parents employed in agriculture | -0.75  (-0.88, -0.62) | -0.36  (-0.51, -0.21) | 0.85  (0.82, 0.88) | 0.90  (0.86, 0.94) | 0.93  (0.89, 0.98) | 0.90  (0.86, 0.95) | 0.32  (0.27, 0.38) | 0.68  (0.55, 0.85) | 0.99  (0.97, 1.02) | 1.01  (0.98, 1.03) |

^1^ All models accounted for representativeness. SEs were clustered at the primary sapling unit level. Adjusted estimates controlled for child age and sex, maternal age and education, paternal age and education, household size, wealth, and location (urban vs. rural). Abbreviations used: MD, mean difference; RR, relative risk
