# Supplementary material for: Maternal and paternal employment in agriculture and early childhood development: A cross-sectional analysis of Demographic and Health Survey data
Source: PLOS Glob Public Health. 2023 Jan 6;3(1):e0001116. doi: 10.1371/journal.pgph.0001116 (PMC10021554; doi:10.1371/journal.pgph.0001116)
Supplement: S7 Table — (DOCX) [file pgph.0001116.s007.docx]

**S7 Table** Associations between parental occupation and childcare practices among children aged 36-59 months^1^

|  | Number of stimulation activities provided by the mother | | Number of stimulation activities provided by the father | | Number of stimulation activities provide by other household members | |
| --- | --- | --- | --- | --- | --- | --- |
|  | Unadjusted MD | Adjusted MD | Unadjusted MD | Adjusted MD | Unadjusted MD | Adjusted MD |
| Both parents employed in agriculture | Ref | Ref | Ref | Ref | Ref | Ref |
| Mother employed in agriculture; father employed in non-agriculture | 0.34 (0.19, 0.49) | 0.17 (0.02, 0.32) | 0.18 (0.06, 0.18) | 0.05 (-0.07, 0.17) | -0.16 (-0.33, 0.00) | -0.18 (-0.34, -0.01) |
| Mother employed in non-agriculture; father employed in agriculture | 0.04 (-0.1, 0.18) | 0.02 (-0.11, 0.16) | 0.06 (-0.04, 0.16) | 0.04 (-0.07, 0.14) | -0.24 (-0.4, -0.08) | -0.27 (-0.43, -0.11) |

|  | Child not left alone for >1 hour in the past week | | Child not left under the supervision of another child for >1 hour in the past week | | Child provided adequate stimulation | | Child attended an early childhood education programme | |
| --- | --- | --- | --- | --- | --- | --- | --- | --- |
|  | Unadjusted RR | Adjusted RR | Unadjusted RR | Adjusted RR | Unadjusted RR | Adjusted RR | Unadjusted RR | Adjusted RR |
| Both parents employed in agriculture | Ref | Ref | Ref | Ref | Ref | Ref | Ref | Ref |
| Mother employed in agriculture; father employed in non-agriculture | 1.01 (0.97, 1.06) | 0.99 (0.95, 1.03) | 1.06 (0.99, 1.13) | 1.04 (0.98, 1.11) | 1.06 (0.99, 1.14) | 1.03 (0.96, 1.11) | 1.77 (1.44, 2.18) | 1.43 (1.18, 1.75) |
| Mother employed in non-agriculture; father employed in agriculture | 0.94 (0.9, 0.98) | 0.95 (0.9, 0.987) | 1.13 (1.06, 1.2) | 1.12 (1.06, 1.19) | 1.08 (1.01, 1.16) | 1.08 (1.01, 1.15) | 2.26 (1.9, 2.69) | 2.07 (1.76, 2.44) |

^1^ All models accounted for representativeness. SEs were clustered at the primary sapling unit level. Adjusted estimates controlled for child age and sex, maternal age and education, paternal age and education, household size, wealth, and location (urban vs. rural). Abbreviations used: MD, mean difference; RR, relative risk
