# Supplementary material for: Maternal and paternal employment in agriculture and early childhood development: A cross-sectional analysis of Demographic and Health Survey data
Source: PLOS Glob Public Health. 2023 Jan 6;3(1):e0001116. doi: 10.1371/journal.pgph.0001116 (PMC10021554; doi:10.1371/journal.pgph.0001116)
Supplement: S8 Table — (DOCX) [file pgph.0001116.s008.docx]

**S8 Table** Associations between parental occupation and women’s empowerment among children aged 36-59 months^1^

|  | Access | | Decision making | | Wife beating | | Empowerment | |
| --- | --- | --- | --- | --- | --- | --- | --- | --- |
|  | Unadjusted MD | Adjusted MD | Unadjusted MD | Adjusted MD | Unadjusted MD | Adjusted MD | Unadjusted MD | Adjusted MD |
| Both parents employed in agriculture | Ref | Ref | Ref | Ref | Ref | Ref | Ref | Ref |
| Mother employed in agriculture; father employed in non-agriculture | 0.00  (-0.05, 0.06) | -0.03  (-0.08, 0.03) | 0.07  (0.01, 0.07) | -0.01  (-0.06, 0.05) | -0.01  (-0.16, 0.13) | -0.06  (-0.21, 0.09) | 0.06  (-0.12, 0.23) | -0.10  (-0.27, 0.07) |
| Mother employed in non-agriculture; father employed in agriculture | 0.43  (0.37, 0.50) | 0.42  (0.36, 0.49) | -0.01  (-0.06, 0.05) | 0.02  (-0.04, 0.07) | 0.73  (0.59, 0.88) | 0.67  (0.53, 0.82) | 1.16  (0.97, 1.35) | 1.11  (0.92, 1.30) |

^1^ All models accounted for representativeness. SEs were clustered at the primary sapling unit level. Adjusted estimates controlled for child age and sex, maternal age and education, paternal age and education, household size, wealth, and location (urban vs. rural). Abbreviations used: MD, mean difference
