# Supplementary material for: Maternal and paternal employment in agriculture and early childhood development: A cross-sectional analysis of Demographic and Health Survey data
Source: PLOS Glob Public Health. 2023 Jan 6;3(1):e0001116. doi: 10.1371/journal.pgph.0001116 (PMC10021554; doi:10.1371/journal.pgph.0001116)
Supplement: S9 Table — (DOCX) [file pgph.0001116.s009.docx]

**S9 Table** Heterogeneity of the associations between parental occupation, child development, childcare practices, and women’s empowerment by household location, household wealth, parental education, and country income level comparing both parents employed in agriculture vs. both parents employed in non-agriculture^1^

|  | Overall development on-track (N=7,270) Adjusted RR (95% CI) | Number of stimulation activities provided by the mother (N=7,270) Adjusted MD (95% CI) | Number of stimulation activities provided by the father  (N=7,270) Adjusted MD (95% CI) | Number of stimulation activities provided by other household members (N=7,270) Adjusted MD (95% CI) | Child provided adequate supervision (N=7,270) Adjusted RR (95% CI) | Child attended an early childhood education programme (N=7,270) Adjusted RR (95% CI) | Total empowerment score (N=7,270) MD (95% CI) |
| --- | --- | --- | --- | --- | --- | --- | --- |
| Household location |  |  |  |  |  |  |  |
| Urban | 0.88  (0.70, 1.11) | 0.47  (-0.04, 0.98) | 0.11  (-0.27, 0.48) | -0.21  (-0.61, 0.19) | 0.87  (0.70, 1.09) | 0.47  (0.26, 0.85) | -0.43  (-0.85, -0.01) |
| Rural | 0.86  (0.79, 0.92) | -0.16  (-0.32, 0.00) | -0.05  (-0.18, 0.08) | 0.31  (0.13, 0.49) | 0.82  (0.77, 0.88) | 0.45  (0.38, 0.53) | -1.06  (-1.24, -0.88) |
| p-value for interaction | 0.79 | 0.02 | 0.43 | 0.02 | 0.62 | 0.91 | 0.01 |
| Household wealth |  |  |  |  |  |  |  |
| Poorest | 0.91  (0.77, 1.08) | -0.22  (-0.5, 0.07) | -0.12  (-0.4, 0.16) | 0.52  (0.15, 0.90) | 0.87  (0.75, 1.00) | 0.43  (0.28, 0.65) | -0.57  (-0.99, -0.16) |
| Poorer | 0.94  (0.82, 1.08) | -0.14  (-0.41, 0.13) | -0.27  (-0.65, 0.10) | 0.09  (-0.39, 0.57) | 0.91  (0.81, 1.01) | 0.53  (0.39, 0.74) | -0.72  (-1.06, -0.38) |
| Middle | 0.82  (0.72, 0.94) | 0.07  (-0.21, 0.34) | 0.17  (-0.05, 0.39) | 0.19  (-0.08, 0.47) | 0.74  (0.66, 0.84) | 0.43  (0.31, 0.60) | -1.14  (-1.49, -0.79) |
| Richer | 0.77  (0.67, 0.89) | 0.09  (-0.20, 0.39) | 0.07  (-0.17, 0.32) | 0.07  (-0.29, 0.42) | 0.76  (0.66, 0.88) | 0.45  (0.33, 0.61) | -1.20  (-1.52, -0.88) |
| Richest | 0.85  (0.70, 1.03) | -0.21  (-0.64, 0.22) | 0.05  (-0.33, 0.43) | -0.08  (-0.55, 0.4) | 0.86  (0.70, 1.06) | 0.59  (0.38, 0.89) | -1.30  (-1.76, -0.83) |
| p-value for interaction | 0.29 | 0.36 | 0.35 | 0.27 | 0.10 | 0.68 | 0.03 |
| Maternal education |  |  |  |  |  |  |  |
| No education | 0.80  (0.71, 0.89) | 0.10  (-0.13, 0.32) | 0.11  (-0.05, 0.27) | 0.30  (0.04, 0.55) | 0.81  (0.73, 0.90) | 0.33  (0.24, 0.45) | -1.16  (-1.41, -0.91) |
| Primary | 0.87  (0.80, 0.95) | -0.08  (-0.27, 0.12) | -0.05  (-0.19, 0.1) | 0.27  (0.07, 0.48) | 0.83  (0.76, 0.91) | 0.54  (0.44, 0.66) | -0.89  (-1.12, -0.65) |
| Secondary or higher | 0.98  (0.85, 1.13) | -0.42  (-0.78, -0.05) | -0.25  (-0.65, 0.16) | 0.07  (-0.37, 0.51) | 0.87  (0.75, 1.00) | 0.39  (0.27, 0.56) | -0.93  (-1.25, -0.61) |
| p-value for interaction | 0.07 | 0.04 | 0.11 | 0.65 | 0.75 | 0.02 | 0.24 |
| Paternal education |  |  |  |  |  |  |  |
| No education | 0.75  (0.66, 0.84) | 0.05  (-0.20, 0.31) | 0.10  (-0.03, 0.23) | 0.35  (0.08, 0.61) | 0.80  (0.71, 0.90) | 0.36  (0.25, 0.52) | -1.16  (-1.47, -0.85) |
| Primary | 0.90  (0.82, 0.98) | 0.02  (-0.18, 0.22) | 0.04  (-0.12, 0.19) | 0.26  (0.04, 0.48) | 0.84  (0.77, 0.91) | 0.50  (0.41, 0.62) | -0.92  (-1.15, -0.70) |
| Secondary or higher | 0.89  (0.78, 1.00) | -0.32  (-0.58, -0.07) | -0.22  (-0.53, 0.09) | 0.15  (-0.19, 0.49) | 0.86  (0.77, 0.96) | 0.48  (0.36, 0.64) | -1.02  (-1.30, -0.75) |
| p-value for interaction | 0.03 | 0.05 | 0.15 | 0.65 | 0.68 | 0.28 | <0.01 |
| Country income level |  |  |  |  |  |  |  |
| Low income | 0.83  (0.77, 0.90) | -0.03  (-0.22, 0.15) | 0.15  (-0.01, 0.30) | 0.42  (0.21, 0.63) | 0.82  (0.76, 0.89) | 0.52  (0.44, 0.63) | -0.33  (-0.52, -0.14) |
| Lower-middle income | 1.01  (0.91, 1.13) | -0.12  (-0.36, 0.13) | -0.24  (-0.41, -0.07) | 0.07  (-0.19, 0.34) | 1.00  (0.92, 1.09) | 0.36  (0.26, 0.51) | -1.14  (-1.41, -0.87) |
| p-value for interaction | <0.01 | 0.58 | <0.01 | 0.04 | <0.01 | 0.05 | <0.01 |

^1^ All models accounted for representativeness. SEs were clustered at the primary sapling unit level. Adjusted estimates controlled for child age and sex, maternal age and education, paternal age and education, household size, wealth, and location (urban vs. rural). Abbreviations used: MD, mean difference; RR, relative risk
