# Supplementary material for: Maternal and paternal employment in agriculture and early childhood development: A cross-sectional analysis of Demographic and Health Survey data
Source: PLOS Glob Public Health. 2023 Jan 6;3(1):e0001116. doi: 10.1371/journal.pgph.0001116 (PMC10021554; doi:10.1371/journal.pgph.0001116)
Supplement: Attachment — Submitted filename: R1. Response to reviewers.docx [file pgph.0001116.s010.docx]

We thank the reviewers for their time and careful consideration of our manuscript. We have incorporated their suggestions, which we believe have improved the paper. We provide point-by-point responses below highlighting changes made in the text. Line numbers refer to the clean version of the manuscript (without tracked changes).

**REVIEWER 1**

**The study reports on maternal and paternal employment in agriculture and early childhood development from DHS data of 10 countries. The study concludes that parental agricultural employment was associated with poorer child development. The study is well conceptualised and written. However, before concluding that it is the occupation of agriculture, which a risk factor, some issues need elucidation.**

We thank the reviewer for their support of our paper. We have addressed their comments in the text and provide point-by-point responses below.

**Major issues:1. All the countries reported (9 from Sub-Saharan Africa and Cambodia) have high levels of child stunting, wasting, anaemia and low birth weight. Since development is intimately linked to child growth and nutrition, it is likely that children from the agriculturists' families (which are poorer) are likely to have greater malnutrition. Child growth indicator data is available through DHS & can be incorporated in the analytic framework.**

Thank you for raising this point. Including child growth was beyond the scope of our study for three main reasons.

1. Although child development and child growth are correlated, there is no evidence that this relationship is causal (Leroy et al. 2020). Thus, we would have had to include child growth as an outcome alongside child development. As we summarize in the Introduction (section 1.1, lines 37-62), there is already extensive literature on the associations between maternal employment and child growth in LMICs. We have provided additional references to further emphasize how well studied this relationship is (Abbi et al., 1991; Glick & Sahn, 1998; Mugo, 2012; Rashad & Sharaf, 2019), see manuscript for full citations). Including child growth as an outcome in our analysis would have made limited contribution to the literature. The more important contribution of our analysis is showing an association between parental employment/occupation and child development, which has not been previously shown.
2. There is no evidence that the childcare mechanisms that we conceptualized as underlying the associations with child development are related to child growth outcomes. For example, maternal engagement in stimulation is not associated with child growth. Thus, we would have had to limit our analysis to women’s empowerment as the single common mechanism linked to both child development and child growth. Again, there is extensive literature on the role of women’s empowerment in child growth (Santoso et al. 2019), but only a limited, emerging literature on the role of women’s empowerment in child development.
3. Our analyses included children 36-59 months of age. There is limited to no evidence of interventions that can effectively improve child *linear* growth in this age group. In contrast, child development is highly malleable in this age group and many interventions have been shown to improve child development (Jeong et al. 2021). Since our goal was to help inform the design of interventions, we focused on outcomes that can more easily be nudged among children 36-59 months of age.

In summary, pathway A in the figure below has already been explored extensively in the literature. Our paper is amongst the first to explore pathway B. Increasing evidence supports that pathway C is not causal (Leroy et al. 2020), i.e., child growth and development are associated, and this association is not causal. Therefore, child growth should not be included in a causal framework regarding employment in agriculture and child development.

Leroy JL, Frongillo EA, Dewan P, Black MM, Waterland RA. Can Children Catch up from the Consequences of Undernourishment? Evidence from Child Linear Growth, Developmental Epigenetics, and Brain and Neurocognitive Development. Adv Nutr. 2020;11:1032–41.

Santoso M V, Kerr RB, Hoddinott J, Garigipati P, Olmos S, Young SL. Role of Women’s Empowerment in Child Nutrition Outcomes: A Systematic Review. Adv Nutr. 2019;

Jeong J, Franchett EE, Ramos de Oliveira C V., Rehmani K, Yousafzai AK. Parenting interventions to promote early child development in the first three years of life: A global systematic review and meta-analysis. Persson LÅ, editor. PLOS Med. 2021;18:e1003602.

**2. Dietary data, particularly diversity, adequacy and meal frequency can be extracted from DHS.** **It is likely that the agriculturists' families have a greater reliance on staple food cultivation and consumption with poor micronutrient and protein availability. This could help explain the study findings better.**

Thank you for this suggestion. We did not include child diet in the analysis for three reasons.

1. A recent paper using DHS examined the association between child diet and child development in children 36-59 months of age (same age group as in our paper) and showed that child diet is not associated with child development (Bliznashka et al. 2021).
2. Diet diversity, adequacy and meal frequency are indicators validated for children 6-24 months of age (WHO 2021). Although validation efforts are underway (personal communication), currently, there are no diet diversity or quality indicators validated for use among children 36-59 months of age in LMICs. To the best of our knowledge, only one study to-date has evaluated the performance of dietary diversity score (the WHO seven food group score) in predicting micronutrient adequacy in rural Burkinabe children aged 24-59 months of age (Diop et al. 2021). This same dietary diversity indicator was used by the above-mentioned study by Bliznashka et al. which found no relationship between child diet and development in children 36-59 months of age using DHS data.
3. Diet data are collected for a sub-sample of children aged 36-59 months. Since this sub-sample with available diet data does not fully overlap with our sample (which was children with child development data both of whose parents were interviewed), including it would have substantially reduced the analytic sample size.

For these reasons we did not include child diet in the analysis. However, the lack of empirical support for a link between child diet and development may be due to numerous data limitations, one of which we have outline in point 2 above (more outlined in the cited article). It may also be because the association was examined concurrently. We agree with the reviewer that child diet may still be an important mechanism to explore in the future, particularly child diet in early infancy, and we have noted this in the Discussion (lines 451-456): *“Other early life biological and nutritional mechanisms beyond our proposed framework could also be explored. For example, evidence suggests that better diet in early infancy predicts improved development later in childhood [60,61]. It is likely that families where both parents are engaged in agriculture rely more on staple foods, resulting in poor micronutrient and protein intake for themselves and their children. However, we lacked data on dietary intake in early childhood and were therefore unable to explore this mechanism.”*

Bliznashka L, Perumal N, Yousafzai A, Sudfeld C. Diet and development among children aged 36–59 months in low-income countries. Arch Dis Child. 2021;archdischild-2021-323218.

World Health Organization, United Nations Children’s Fund. Indicators for assessing infant and young child feeding practices: definitions and measurement methods. Geneva:; 2021.

Diop L, Becquey E, Turowska Z, Huybregts L, Ruel MT, Gelli A. Standard Minimum Dietary Diversity Indicators for Women or Infants and Young Children Are Good Predictors of Adequate Micronutrient Intakes in 24–59-Month-Old Children and Their Nonpregnant Nonbreastfeeding Mothers in Rural Burkina Faso. J Nutr. 2021;151:412–22.

**Minor issues:**

**1. The authors do mention about pesticide exposure and its possible adverse effects. They understandably cannot report on it because of lack of data. However, tobacco use (smokeless as well as smoked) is widely prevalent in LMICs especially in the rural populations. A significant exposure to smokeless tobacco use is reported in even pregnant women in LMICs. This could adversely affect parental health and child development.**

Thank you for this suggestion. We agree that substance use (tobacco and alcohol) is important to consider and have now commented on it in the Discussion on lines 482-489: *“Future work should also consider additional factors like parental use of alcohol, tobacco, and nicotine-containing products, which is prevalent in LMICs [64] and in farming populations [65], and associated with child development, particularly with poor emotional development and behavioural difficulties [66–68]. More evidence is needed on if, and how, parental substance use influences the relationship between parental occupation and child development in LLMICs. Due to data limitations on parental substance use and the fact that ECDI does not assess emotional development or behaviour, we did not include substance use in our analysis.”*

With respect to data limitations, one of the countries in our sample (Senegal) did not collect data on tobacco and cigarette use by men and women. In the remaining countries in our sample, limited data on tobacco and cigarette use among men and women was collected, which made it difficult to harmonize indicators. The prevalence of cigarettes and tobacco use among men and women was also very low in some countries in our sample, such as Togo. All of these data limitations made it difficult to adequately assess the role of tobacco and cigarette use in explaining the associations we examined. Since the main analysis we conducted pooled data from all countries, by including a variable not measured in one country, the main analysis would exclude that country entirely. Therefore, we chose not to include tobacco use in the analysis.

**2. Alcohol use is also having significant prevalence among the rural poor in LMICs. This could be important in the context of wife beating, access and decision making, measured for women's empowerment, in the study.**

Please see previous response. With respect to data limitations on alcohol use, some of the DHS surveys in our sample only collected data on whether the husband/partner drinks alcohol and whether alcohol was consumed the last time they were sexually active. Some countries like Benin did not collect data on women’s alcohol use. These data limitations made it difficult to harmonize the data and adequately examine the role of parental alcohol consumption in explaining the associations we investigated. Similar to tobacco use, if we were to include alcohol use which was not measured in all country, the main analysis would entirely exclude those countries where the variable was not measured. Therefore, we chose not to include alcohol use in the analysis.

**3. Use of mosquito nets: many of these countries have endemicity of Malaria which is known to affect younger children far more than other age groups. Anemia as a consequence of Malaria could well pull down development scores. Use of mosquito nets is recorded in DHS and can be included in this analysis.**

Thank you for raising this point. We agree that malaria and anaemia are important risk factors for child development. We considered including an indicator for whether the child slept under an insecticide-treated bednet the night before the survey. However, this variable was not available for one of the countries in our sample (Cambodia). As mentioned above, including a variable not measured in one country would exclude that country from the analysis. We therefore did not control for it in our analyses. Further, there is little evidence that whether the child slept under a bednet is a confounder of the association between parental employment and child development. In fact, in our sample, there did not appear to be any substantive differences in bednet use by parental employment (see tabulation below).

```
     Child |
     slept |
   under a |
   treated |
    bednet |  Mother is employed
last night |        No        Yes |     Total
-----------+----------------------+----------
        No |       547      3,425 |     3,972
           |     48.41      51.05 |     50.67
-----------+----------------------+----------
       Yes |       583      3,284 |     3,867
           |     51.59      48.95 |     49.33
-----------+----------------------+----------
     Total |     1,130      6,709 |     7,839
           |    100.00     100.00 |    100.00

     Child |
     slept |
   under a |
   treated |
    bednet |             Parental occupation
last night | Mother in  Mother in  Both in a  Both in n |     Total
-----------+--------------------------------------------+----------
        No |       414        601      1,557        853 |     3,425
           |     49.58      52.03      55.27      44.85 |     51.05
-----------+--------------------------------------------+----------
       Yes |       421        554      1,260      1,049 |     3,284
           |     50.42      47.97      44.73      55.15 |     48.95
-----------+--------------------------------------------+----------
     Total |       835      1,155      2,817      1,902 |     6,709
           |    100.00     100.00     100.00     100.00 |    100.00
```

**4. Lastly, since it is a multi-country study, there are likely to be significant differences between countries with respect to policies and provision of care like ECCE or health care facilities for women and children. This could be pertinent in the context of nutritional interventions, malaria, anaemia, tuberculosis or such tropical illnesses which have a wide impact on child development and growth. These differences can be mentioned in the study.**

Thank you for raising this point. We agree that there are important differences across countries in the provision of maternal and child health, nutrition, and development services as well as in the national and sub-national policies and strategies to improve child wellbeing. We have now included heterogeneity analysis by country income level (low income vs. lower-middle income). We found that country income level modified all associations we examined except for the association between parental occupation and number of stimulation activities provided by the mother. We have updated the methods (line 246), results (Section 3.5, lines 335-368), and associated figures and tables. For brevity, we refer the reviewer to the manuscript. We also discuss these findings on lines 471-478:

*“Finally, country income level modified all associations we examined except for the association between parental agricultural employment and number of stimulation activities provided by the mother. However, patterns were inconsistent here as well with less beneficial associations for child development and adequate supervision in low income countries and for number of stimulation activities provided by the father, ECCE attendance, and women’s empowerment in lower-middle income countries. Country income level likely reflects various country-level characteristics that influence these associations, such as the size of the agricultural sector, access to and quality of healthcare and early childhood care and education services, and gender equity…..* *Future studies should examine other country-level characteristics that may moderate the associations we examined.”*

**REVIEWER 2**

**Summary

The objective of this primary research article was to determine the association between parental employment type and child development, and explore childcare practices and women's empowerment as potential mechanisms. 9 DHS surveys, primarily from Africa, were pooled for the analysis. Parental agricultural employment, as opposed to non-agricultural employment, was found to be a risk factor for child development outcomes, exposure to cognitive stimulation, attendance of early care and education services, inadequate supervision, and women's empowerment.**

**While the paper highlights the gaps in the literature and explains the rationale of the study very clearly, my main concern is related to the interpretation of the results. The authors have demonstrated that agricultural employment is more likely to be practiced by poorer, less educated individuals, who typically reside in rural areas. Therefore, one can argue that it is poverty, material deprivation, and the lack of adequate cognitive stimulation, rather than the type of employment, that is really driving these associations. Are similar results seen in populations where agricultural employment is practiced by more affluent and educated families?**

Thank you for raising this important point. We have reflected on it in the Discussion on lines 523-535: *“Relatedly, we could not resolve issues of endogeneity inherent in cross-sectional samples. Specifically, we could not determine the direction of the relationship between parental occupation type and parental education, household wealth, and household location. For example, we could not disentangle whether poorer parents chose to work in agriculture or whether those working in agriculture remained poorer. As a result, given that parental occupation type differed across parental education, As a result, given that parental occupation type differed across parental education, household wealth, and household location, we were unable to conclusively demonstrate that the associations we observed were driven by parental occupation rather than by these other factors. We controlled for these factors in the adjusted models, which helped minimize confounding. However, we cannot rule out the presence of residual confounding. Replication in samples where more affluent and educated families engage in agriculture and less affluent and less educated families engage in non-agriculture is needed to provide more definitive support for our hypotheses.”*

**Abstract:

The following statement is not valid - Our objective was to investigate the associations between maternal and paternal agricultural employment - since all types of employment were considered for analysis, not just agricultural employment. This statement is therefore biased towards which of the results showed significant differences.**

We have restated the objective to better align with our analyses (lines 6-8): *“Our objective was to investigate the associations between maternal and paternal employment (comparing agricultural and non-agricultural employment) and child development and to examine childcare practices and women’s empowerment as potential mechanisms.”*

**Introduction:

The hypothesized mechanisms by which parental occupation type may affect child development outcomes are very well laid out. However, some of them were not tested (household income, parental physical and mental health). While the reason for not being able to examine pesticides exposure as a mechanism is indicated as a limitation later on, the reasons for leaving out the ones mentioned in the parenthesis are not clear. More so since data on both health and income are comprehensively collected in the DHS surveys.**

Thank you for raising this point. We have expanded on it in the Discussion on lines 435-451: *“We did not investigate household income, parental physical and mental health, and pesticide exposure as mechanisms in our analysis due to lack of data. First, DHS surveys do not collect data on household income. Instead, the DHS Program calculates a household wealth index representing fixed assets that cannot easily be converted into resources for child development. Second, with respect to parental physical and mental health, DHS surveys collect data on maternal perinatal health with respect to the most recent pregnancy but do not collect data on current/general physical health, which is likely a more viable mechanism between parental employment and child development. A maternal mental health module was introduced in DHS Phase 8 in 2020 and thus not available for the surveys in our sample. Data on paternal physical and mental health is also lacking. Third, with respect to pesticide exposure, DHS surveys do not collect data on agricultural or occupational pesticide exposure. However, given the wide use of pesticides in LLMICs* (FAO, 2021) *and pesticides’ direct adverse effects on child development through acetylcholinesterase inhibition* (González-Alzaga et al., 2014; Kwong, 2002; Sapbamrer & Hongsibsong, 2019)*, we cannot rule out that pesticide exposure was a major contributor towards the poorer child development outcomes we observed among agricultural families relative to non-agricultural families. Future work should collect data on these mechanisms to better understand their contribution towards child development and to help design interventions that support children and their families.”*

**The literature review is very well done. It flows well into the stated objectives.**

Thank you!

**Methods:

I am curious to know why the DHS surveys from these nine countries were specifically chosen for this study. Since this is a secondary data analysis project, why not include DHS data from all LLMICs surveys?**

Thank you for raising this point. We have clarified in the Methods on lines 159-166: *“Since the child development module is optional, we could only include surveys which collected this module. However, within a DHS survey, the sub-samples of households completing the child development module and the man’s interview are not always overlapping. Therefore, not all DHS surveys collecting data on child development contributed data to our analysis. In total, we included nine DHS spanning 2011-2020: Benin, Burundi, Cambodia, Congo, Haiti, Rwanda, Senegal, Togo, and Uganda (Supplemental Table 1).* *These were all the countries with an overlapping sub-sample of households with data on child development and paternal employment.”*

**Given my concern above (poverty and material deprivation, rather than occupation type, is driving child development outcomes), can the authors conclusively demonstrate / prove their hypotheses? Could they consider a propensity score matching type of statistical method to resolve this?**

Please see our response above related to your concern about poverty and material deprivation. Regarding your second point, we did not consider statistical methods like propensity score matching given the largely exploratory nature of our analysis. Our objective was to assess the association between occupation type (using the crude indicators available in the DHS) and child development rather than to identify an “effect” of occupation type on child development (when propensity score matching would have been useful). We believe the issue raised by the reviewer will most adequately be addressed by specifically designing studies to assess the relationship between parental occupation and child development, drawing adequate samples and using more comprehensive occupation indicators. For this reason, we repeatedly note throughout the Discussion that our analysis was hypothesis-generating and further replication is needed.

**Results:

Please clarify lines 290-293: Further, when both parents were employed in agriculture, they were less likely to provide adequate child supervision (particularly, not leave the child with an older sibling) compared to when both parents were employed in non agriculture (Table 4).**

We have revised the sentence and hope it is now clearer (lines 318-320): *“Further, parental agricultural employment relative to non-agricultural employment was associated with inadequate child supervision, particularly leaving the child with an older sibling (Table 4).”*

**Discussion:

In Line 318, add 'surveys' after DHS**

Added

**Line 319: Clarify that the authors intended to investigate the association between parental occupation type and child development...not "agricultural" employment alone**

We have now clarified (lines 369-372): *“In this study, we used nationally representative data from nine DHS surveys to investigate the relationship between parental employment (comparing agricultural and non-agricultural employment) and child development, and the role of childcare practices and women’s empowerment as potential mechanisms.”*

**Hence, overall a very good read, but I am worried about the interpretation. I look forward to a rebuttal which will address my concerns.**

We thank the reviewer for their support of our study. We have incorporated their suggestions, which we believe have improved our paper.
