# Supplementary material for: Maternal and paternal employment in agriculture and early childhood development: A cross-sectional analysis of Demographic and Health Survey data
Source: PLOS Glob Public Health. 2023 Jan 6;3(1):e0001116. doi: 10.1371/journal.pgph.0001116 (PMC10021554; doi:10.1371/journal.pgph.0001116)
Supplement: Attachment — Submitted filename: R2. Response to reviewers.docx [file pgph.0001116.s011.docx]

We thank the reviewers for their time and careful consideration of our manuscript. We have incorporated their suggestions, which we believe have improved the paper. We provide point-by-point responses below highlighting changes made in the text. Line numbers refer to the clean version of the manuscript (without tracked changes).

**REVIEWER 1**

**Reviewer #1: The Leroy et al paper referred to is on the relationship of stunting and child development & not on wasting.1 There is substantial published evidence indicating association between wasting (or acute child malnutrition) and even stunting with child development.2,3 While the authors’ state that there is no evidence that child growth and development are causally related, there is a significant association between the two.**

**Moreover, it is entirely plausible that a growth retarded and wasted child takes longer time to care & feed. There is also a likelihood of developmental delay in such a situation. Hence, the set of variables are complex and interrelated. Also, as per the well-established UNICEF framework for malnutrition, both nutrition and care are contributory to child growth and well-being.4**

**Studying the association between parental employment and child development cannot ignore this other major contributor, namely, malnutrition, as measured through anthropometry. The authors have also included nutrition as one of the agencies through which parental employment changes child development(under household income).**

**This is all the more significant in view of the authors’ conclusions relating agricultural work to child development. It should be noted that much of the food in Africa and Asia is produced by small holder farmers who ironically are the worst affected by malnutrition.5,6**

**It will be appropriate to use malnutrition related variables ,particularly wasting prevalence, as a contributory factor to child development in the analysis.**

**Even while considering both as outcomes of similar variables, it will be preferable to check for multicollinearity of anthropometric variables of various groups of families with respective child development, in the individual(disaggregated) country settings.**

Thank you for these suggestions. We have now included a sensitivity analysis controlling for child wasting in the adjusted models for the associations between maternal employment, parental occupation, and child development. We describe the additional analyses on lines 243-252 of the Methods:

*“Given the role of child nutrition (safe and nutritious foods) in supporting child wellbeing [57] and evidence that severe childhood malnutrition can impair child development [58], we conducted a sensitivity analysis accounting for child wasting (defined as weight-for-height Z-score <-2 SD) in the analysis. We first examined biserial correlations between child wasting, parental employment, and child development in the pooled sample and separately by country. We then included child wasting as a control variable in the adjusted models for the associations between maternal employment, parental occupation, and child development. Of note, child wasting was not available for the sample of children with data on child development and maternal and paternal occupation in Cambodia and Congo. Therefore, these sensitivity analyses were conducted on a dataset pooling the remaining seven countries.”*

We present correlations on lines 284-289 of the Results: *“Overall, 2.4% of children in our sample were wasted, ranging from 0.6% in Rwanda to 10% in Senegal. In the pooled sample, child wasting was significantly correlated with maternal employment (p<0.01); however, the magnitude of this correlation was small (-0.03). Child wasting was not correlated with child development (overall or by domain) in our sample (all p-values >0.3 and all correlations were between -0.01 and 0.01). Results were similar by country (data not shown).”*

We note in the Results that findings on the associations between maternal employment, parental occupation, and child development are consistent between the models controlling and not controlling for child wasting:

*“Results were consistent in sensitivity analyses controlling for child wasting (RR 1.01 (95% CI 0.95, 1.08)).” (lines 296-297)*

*“Results were consistent in sensitivity analyses controlling for child wasting (RR 0.85 (95% CI 0.79, 0.92)).” (lines 314-315)*

We discuss implications and future directions on lines 480-490 of the Discussion: *“We considered the role of child wasting in sensitivity analyses and found no evidence that child wasting influenced the associations between maternal employment, parental occupation, and child development. This may be due to the low prevalence of child wasting in our sample (2.4%) or because undernutrition may be less harmful in children 36-59 months of age than in younger children [64]. Further, a growing body of evidence suggests that malnutrition in early childhood impairs cognitive, academic, and human capital outcomes later in childhood and adulthood [58,65–67]. Due to the lack of data, we could only assess the role of concurrent child wasting. Although we found no evidence of a cross-sectional association between child wasting and development, future studies should consider previous and persistent episodes of child malnutrition, and if and how they may influence the associations between parental employment and child development.”*

**Line 344-349 sentence is too unwieldy and long.**

We have revised the sentence (lines 362-368): *“There was also evidence that household location (urban vs. rural), parental education, and country income level modified the associations between occupation and stimulation (p-values for interaction <0.10). We observed less beneficial associations for stimulation by the mother among rural households and more educated parents, a less beneficial association for stimulation by the father in lower-middle income countries, and a more beneficial association for stimulation by other household members among rural households and in low income countries (****Figure 2****, Supplemental Table 9).”*

**REVIEWER 2**

**1. Regarding the author's point: First, DHS surveys do not collect data on household income. Instead, the DHS Program calculates a household wealth index representing fixed assets that cannot easily be converted into resources for child development.**

**- While I agree that household assets are not a good reflection of resources for child development, one could argue that they are a proxy measure, since those with higher number of household assets may also be able to afford more resources related to children's development.**

**Please can I request the authors to discuss why they may be seeing a bigger difference between agricultural vs non-agricultural employment in the richer/richest households compared to the poorest households with respect to "on-track" development. It would also help to highlight the sample size in each of these categories. Eg: how many families have both parents employed in the agricultural sector in the richest vs poorest categories. I just want to make sure that small numbers are not leading to misinterpretation of the results.**

Thank you for raising this point. We have noted the proportion of parents engaged in agricultural and non-agricultural employment by household wealth in the main text (lines 273-275): *“Both parents were engaged in agriculture in 35% of the poorest households and 3% of the wealthiest households. Both parents were engaged in non-agriculture in 8% of the poorest households and 38% of the wealthiest households (Supplemental Table 3).”*

We discuss the implications on lines 562-571: *“As a result, given that parental occupation type differed across parental education, household wealth, and household location, we were unable to conclusively demonstrate that the associations we observed were driven by parental occupation rather than by these other factors. We controlled for these factors in the adjusted models, which helped minimize confounding. However, we cannot rule out the presence of residual confounding. In addition, small sample sizes in sub-groups of households may have biased the results: both parents were engaged in agriculture in 3% of the wealthiest households, whereas both parents were engaged in non-agriculture in 8% of the poorest households. Replication in larger samples where more affluent and educated families are engaged in agriculture and less affluent and less educated families are engaged in non-agriculture is needed to provide more definitive support for our hypotheses.”*
